# Clinicopathological and Genomic Characterization of a Simmental Calf with Generalized Bovine Juvenile Angiomatosis

**DOI:** 10.3390/ani11030624

**Published:** 2021-02-26

**Authors:** Joana G. P. Jacinto, Irene M. Häfliger, Nicole Borel, Patrik Zanolari, Cord Drögemüller, Inês M. B. Veiga

**Affiliations:** 1 Department of Veterinary Medical Sciences, University of Bologna, 40064 Ozzano Emilia (BO), Italy; joana.goncalves2@studio.unibo.it; 2 Institute of Genetics, Vetsuisse Faculty, University of Bern, 3012 Bern, Switzerland; irene.haefliger@vetsuisse.unibe.ch; 3 Institute of Veterinary Pathology, Vetsuisse Faculty, University of Zurich, 8057 Zurich, Switzerland; n.borel@access.uzh.ch; 4 Clinic for Ruminants, Vetsuisse Faculty, University of Bern, 3012 Bern, Switzerland; patrik.zanolari@vetsuisse.unibe.ch; 5 Institute of Animal Pathology, Vetsuisse Faculty, University of Bern, 3012 Bern, Switzerland; ines.veiga@vetsuisse.unibe.ch

**Keywords:** cattle, bovine juvenile angiomatosis, vascular hamartoma, precision medicine, vascular malformation, rare diseases, whole-genome sequencing

## Abstract

**Simple Summary:**

Vascular anomalies represent a heterogeneous group of rare disorders encompassing both vascular malformations and tumors, which can be congenital or arise shortly after birth. They often pose a diagnostic challenge in human and veterinary medicine, and the referring nomenclature is equivocal. Bovine juvenile angiomatosis (BJA), a clinical condition belonging to this group of disorders, encompasses vascular malformations and tumors arising in calves. Usually, such vascular anomalies are not further investigated on a molecular genetic level, mainly because of a lack of resources and diagnostic tools, as well as the low value and short lifespan of the affected animals. Here we report the clinical, pathological, immunohistochemical, and genetic features of a Simmental calf that displayed multiple cutaneous, subcutaneous, and visceral vascular hamartomas compatible with a generalized form of BJA. Whole-genome sequencing identified six coding variants, including four heterozygous variants in the *PREX1, UBE3B, PCDHGA2*, and *ZSWIM6* genes, which occurred only in the BJA-affected calf and were absent in the global control cohort of more than 4500 cattle. Assuming a germline mutation as etiology, one of these variants might be responsible for the vascular malformations identified in this calf.

**Abstract:**

Bovine juvenile angiomatosis (BJA) comprises a group of single or multiple proliferative vascular anomalies in the skin and viscera of affected calves. The purpose of this study was to characterize the clinicopathological phenotype of a 1.5-month-old Simmental calf with multiple cutaneous, subcutaneous, and visceral vascular hamartomas, which were compatible with a generalized form of BJA, and to identify genetic cause for this phenotype by whole-genome sequencing (WGS). The calf was referred to the clinics as a result of its failure to thrive and the presence of multiple cutaneous and subcutaneous nodules, some of which bled abundantly following spontaneous rupture. Gross pathology revealed similar lesions at the inner thoracic wall, diaphragm, mediastinum, pericardium, inner abdominal wall, and mesentery. Histologically, variably sized cavities lined by a single layer of plump cells and supported by a loose stroma with occasional acute hemorrhage were observed. Determined by immunochemistry, the plump cells lining the cavities displayed a strong cytoplasmic signal for PECAM-1, von Willebrand factor, and vimentin. WGS revealed six private protein-changing variants affecting different genes present in the calf and absent in more than 4500 control genomes. Assuming a spontaneous de novo mutation event, one of the identified variants found in the *PREX1, UBE3B, PCDHGA2,* and *ZSWIM6* genes may represent a possible candidate pathogenic variant for this rare form of vascular malformation.

## 1. Introduction

Congenital vascular tumors and malformations are rare anomalies that develop during pregnancy or within the first three months of life [1]. The International Society for the Study of Vascular Anomalies (ISSVA) published an updated classification of these lesions in 2020 that includes genetic and extended histologic findings that came to light since the original ISSVA classification was created in 1996 [2,3]. However, the differentiation between vascular tumors and malformations is not always straightforward [4], and the terminology of vascular anomalies often remains a challenge [2], not only in human, but also in veterinary medicine [5,6,7]. Specifically, calves are known to display several types of vascular anomalies, some of which are congenital [6]. Vascular hamartomas are relatively common and might be found in the mandibular gingiva [7,8,9,10,11], skin [12], heart [13], and lung [14]. Hemangioma is the most frequently reported benign vascular neoplasm in calves [15] and might be localized in the gingiva [15,16], skin [5,17,18], heart [6], or multifocally [17]. In addition, although malignant vascular tumors are rarely described in cattle [18,19], a multifocal hemangiosarcoma was diagnosed in a stillborn calf [20]. In 1990, Watson and Thompson [6] proposed that these different manifestations of single and multiple vascular anomalies in calves should be grouped under the term bovine juvenile angiomatosis (BJA). This condition differs from the so-called bovine cutaneous angiomatosis, which is mostly identified in adult dairy cattle with a mean age of 5.5 years and is characterized by the appearance of mostly single, proliferative cutaneous vascular anomalies that range from hamartomas to hemangiomas [21,22,23,24,25]. It was postulated that bovine cutaneous angiomatosis may be a consequence of exuberant granulation tissue formation, especially due to histologic similarities to lobular capillary hemangioma or granulation tissue-type hemangioma of man [6,21]. Some authors mentioned chromosomal abnormalities as a putative cause for the bovine juvenile angiomatosis in comparison to what has been described in humans with similar vascular lesions [6,26]. However, chromosomal abnormalities or other types of genetic mutations have not been identified in calves affected by BJA [6,26].

Herein, we aimed to characterize the clinical and pathological phenotype of multiple cutaneous, subcutaneous, and visceral vascular hamartomas compatible with a generalized form of BJA in a Simmental calf. In addition, whole-genome sequencing (WGS) was carried out to identify putatively pathogenic variants.

## 2. Materials and Methods

### 2.1. Clinical and Pathological Investigation

A 1.5-month-old female Simmental calf with a body weight of 64 kg was referred to the Clinic for Ruminants at the Vetsuisse Faculty, University of Bern, as a result of poor weight gain and skin lesions. Previous treatment by the referring veterinarian included anthelmintics (Ivermectin 0.2 mg/kg, sc, Ivomec, Biokema SA, Crissier, Switzerland) and antibiotics (Benzylpenicillinum procainum, 30,000 IU/kg, iv, Cobiotic, Virbac AG, Glattburg, Switzerland). The calf was euthanized with an intravenous injection of pentobarbital (pentobarbitalum natricum, 150 mg/kg, iv, Streuli Pharma AG, Uznach, Switzerland) and was subsequently submitted to the Institute of Animal Pathology at the Vetsuisse Faculty, University of Bern, for necropsy and histologic examination. Tissue samples from the subcutaneous and internal nodules, as well as from several inner organs, were immediately collected, fixed in 4% buffered formalin, embedded in paraffin, cut at 4 µm, and stained with haematoxylin and eosin (H&E) for further histologic evaluation. Immunohistochemical (IHC) analysis for platelet endothelial cell adhesion molecule (PECAM-1), von Willebrand factor, smooth muscle actin (SMA), vimentin, and a broad-spectrum cytokeratin marker (MNF116) were performed from one subcutaneous and one mediastinal nodule. For the PECAM-1 IHC, antigen retrieval using pressure cooking (98 °C, 20 min) in basic EDTA buffer (pH 9.0) was performed, and the primary antibody (sc1506, Santa Cruz Biotechnology, Dallas, TX, USA) was incubated for 1 h at room temperature (RT) (1:1000 dilution). For the von Willebrand factor IHC, pressure cooking in citate buffer (pH 6.0, S2031 Agilent Technologies, Santa Clara, CA, USA) was performed for antigen retrieval, and the primary antibody (A0082, Agilent Technologies) was incubated for 40 min (1:100 dilution) at RT. Peroxidase blocking (S2023, Agilent Technologies) was performed prior to primary antibody incubation for 10 min in both cases, followed by incubation with Envision+system HRP rabbit (K4003, Agilent Technologies) for 30 min at RT, labeling with 3,3′-diaminobenzidine (DAB) (K3468, Agilent Technologies) for 10 min, counterstaining with hematoxylin, and mounting. For the SMA IHC, no antigen retrieval was performed, primary antibody incubation (M085, Agilent Technologies) took place for 1 h at RT (1:400 dilution), and the Mach 4 Universal HRP Polymer kit (BRR 4012L, Medite, Dietikon, Switzerland) was used as secondary antibody at RT. Peroxidase blocking and DAB labeling were performed as previously described. For the vimentin IHC, antigen retrieval was performed with Bond Epitope Retrieval Buffer Type 2 (Tris-EDTA pH 9, AR9640 Leica Biosystems, Wetzlar, Germany) for 10 min at 95 °C, and was followed by primary antibody incubation (V9, M0725, Agilent Technologies) for 15 min at RT (1:1000 dilution). For the MNF116 IHC, antigen retrieval was performed with Bond Enzyme 1 (AR9551 Leica Biosystems) for 5 min at 37 °C, followed by primary antibody incubation (M0821, Agilent Technologies) for 15 min at RT (1:400 dilution). In both cases, all further steps were performed using reagents of the Bond Polymer Refine Detection Kit (DS9800, Leica Biosystems), namely peroxidase blocking for 5 min, incubation with rabbit anti-mouse secondary antibody for 8 min at RT, and peroxidase-labelled polymer incubation for 8 min. Finally, slides were developed in DAB/H_2_O_2_ for 10 min, counterstained with hematoxylin, and mounted. Tissue sections from normal haired skin from a cow were used as positive control for the PECAM-1, von Willenbrand, vimentin, and MNF116 IHCs, while a tissue section from the small intestine from a cow was used as positive control for the SMA IHC. Tissue sections from the nodules from the affected calf without primary antibody incubation were used as negative controls.

### 2.2. DNA Sample and Whole-Genome Sequencing

Genomic DNA was isolated from the liver of the calf using the Promega Maxwell RSC DNA system (Promega, Dübendorf, Switzerland). WGS using the Illumina NovaSeq6000 was performed on the genomic DNA of the calf. The sequenced reads were mapped to the ARS-UCD1.2 reference genome, resulting in an average read depth of approximately 17.5× [27], and single-nucleotide variants (SNVs) and small indel variants were called. The applied software and steps to process fastq files into binary alignment map (BAM) and genomic variant call format files were in accordance with the 1000 Bull Genomes Project processing guidelines of run 8 (www.1000bullgenomes.com) [28], except for the trimming, which was performed using fastp [29]. Further preparation of the genomic data had been done according to Häfliger et al. 2020 [30]. In order to find private variants, we compared the genotypes of the affected calf with 496 cattle genomes of various breeds that had been sequenced in the course of other ongoing studies at the Institute of Genetics at the Vetsuisse Faculty, University of Bern, and that are publicly available in the European Nucleotide Archive (SAMEA6528880 is the sample accession number of the affected calf; http://www.ebi.ac.uk/en) (Table S1). The filtered list of remaining variants were further checked for their occurrence in a global control cohort of 4110 genomes of a variety of breeds (1000 Bull Genomes Project run 8; www.1000bullgenomes.com accessed on 15 November 2020) [28]. Integrative Genomics Viewer (IGV) [31] software was used for visual inspection of genome regions containing possible candidate genes.

In order to evaluate possible chromosomal abnormalities, the read depth along all chromosomes was calculated. A sliding window approach was used where 3 different window sizes were executed (10 kb, 200 kb, 500 kb). Using the function bedcov of the program Samtools [32], the output generated was the number of reads within each specified window. Furthermore, coverage plots were produced using the function Manhattan of the package “qqman” in R [33].

### 2.3. Evaluation of the Molecular Consequences of Amino Acid Substitutions

PROVEAN [34], MutPred2 [35], and PredictSNP1 [36] were used to predict the functional consequences of the identified variants on protein.

## 3. Results

### 3.1. Clinical Phenotype

At clinical examination, the calf was alert but moderately reduced in its general body condition. The rectal body temperature was 39.0 °C, the heart rate was 80 beats per minute, and the respiratory rate was 40 beats per minute. Examination of the cardiovascular, respiratory, digestive, and urinary systems did not reveal any abnormalities.

**Figure 1:**
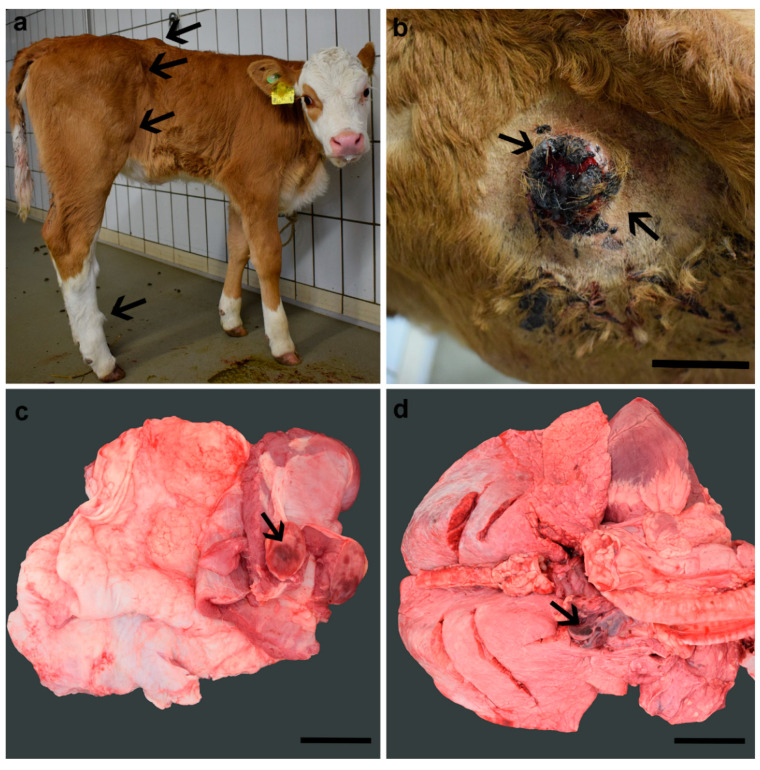
Cutaneous and subcutaneous nodules (arrows) located at the back, flank, and hindlimbs of the Simmental calf (**a**), some of which were covered by abundant serosanguineous crusts (**b**). At necropsy, similar looking nodules (arrows) were identified, namely in the inner thoracic wall (**c**) and in the mediastinum (**d**). All nodules measured up to 5 cm in diameter, were well demarcated from the adjacent tissue, and displayed a white to reddish cut surface. Bar 5 cm (**b**), 3 cm (**c**), and 4 cm (**d**).

The integumentary system of the calf revealed multiple, cutaneous and subcutaneous, soft, movable nodules, which measured up to 5 cm in diameter. These were predominantly present in the caudal aspect of the back, the pelvis, and the hind limbs (Figure 1a). The cutaneous nodules were occasionally covered by sanguineous crusts, and some nodules bled abundantly following spontaneous rupture (Figure 1b). No hematological and biochemical analyses were performed due to cost restrictions. Based on these findings and considering that no other calves in the herd showed similar skin lesions, a presumptive diagnosis of multifocal vascular anomalies was made. The animal was then euthanized due to a lack of response to treatment and poor prognosis.

### 3.2. Pathological Phenotype

At necropsy, the cutaneous and subcutaneous nodules displayed either a white or reddish cut surface. Additionally, mostly pedunculated but similar looking nodules were occasionally present at the inner thoracic wall and diaphragm (Figure 1c), the mediastinum (Figure 1d), the pericardium, the inner abdominal wall, and the mesentery adjacent to the duodenum. The remaining organs were macroscopically unremarkable.

**Figure 2:**
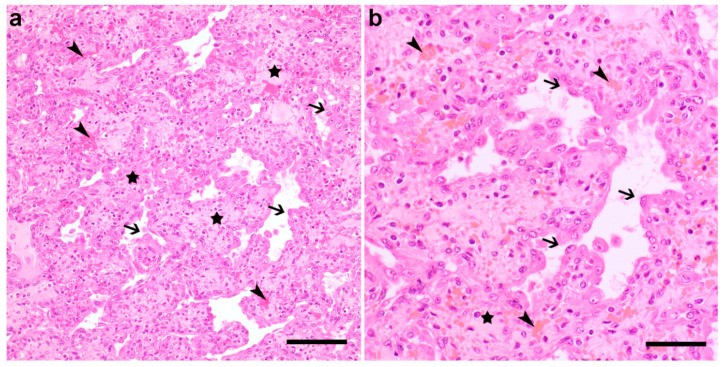
Histology of one of the subcutaneous nodules from the Bovine juvenile angiomatosis (BJA)-affected Simmental calf. Note the variably sized, mostly empty cavities lined by a single layer of plump cells (arrows) and supported by a loose fibrovascular stroma (stars) with occasional acute hemorrhage (arrowheads). Hematoxylin and eosin (H&E) staining, Bar 100 µm (**a**) and 50 µm (**b**).

Histologically, all analyzed nodules consisted of encapsulated (with the exception of the nodule present in the mesentery adjacent to the duodenum), well demarcated, expansively growing, moderately cellular masses. These consisted of abundant, variably sized, mostly empty cavities, which were lined by a single layer of plump cells and supported by a loose, partially edematous fibrovascular stroma (Figure 2a,b), with occasionally intermingled foci of mature connective tissue. The plump cells displayed a moderate amount of eosinophilic, homogeneous cytoplasm, an oval nucleus with finely stippled chromatin, and up to two basophilic, round nucleoli. The anisocytosis and anisokaryosis were low to moderate, and there were very few mitotic figures visible. Moderate to high numbers of free erythrocytes (compatible with acute hemorrhage), as well as occasional neutrophils and lymphocytes, were visible within the stroma.

**Figure 3:**
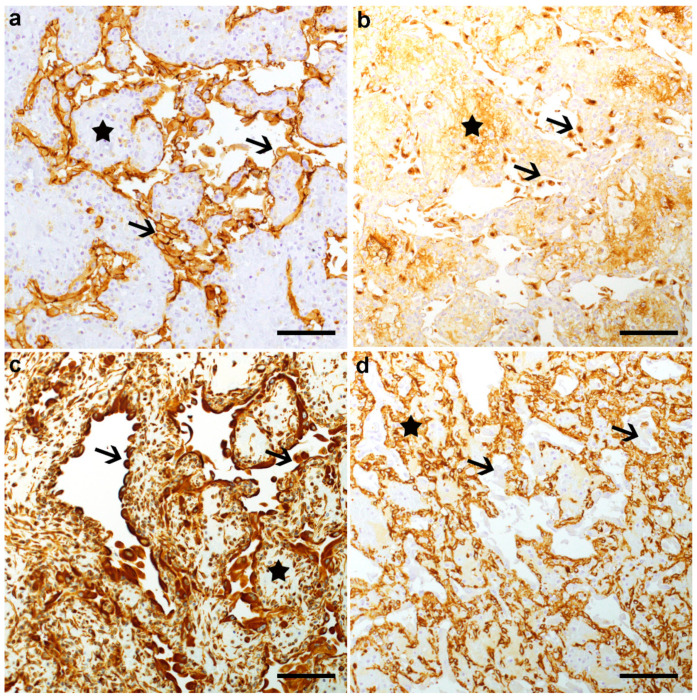
Immunohistochemical (IHC) analysis of a subcutaneous nodule from the BJA-affected Simmental calf with platelet endothelial cell adhesion molecule (PECAM-1) (**a**), von Willebrand factor (**b**), vimentin (**c**), and smooth muscle actin (SMA) (**d**). Note that the plump cells lining the cavities (arrows) displayed a strong positive signal in the PECAM-1, von Willebrand factor, and vimentin IHC but were negative in the SMA IHC. The stromal cells (stars) displayed a positive cytoplasmic signal in the vimentin and SMA IHC staining, while the signal seen in the von Willebrand factor IHC was considered to represent background staining. IHC staining, Hematoxylin counterstain, Bar 100 µm.

To determine the cellular origin of these nodules, IHC was performed. In both nodules, the plump cells lining the cavities displayed a strong cytoplasmic signal for PECAM-1 (Figure 3a), von Willebrand factor (Figure 3b), and vimentin (Figure 3c) but not for SMA (Figure 3d). In addition, the supporting stromal cells displayed a strong cytoplasmic signal for vimentin (Figure 3c) and SMA (Figure 3d), as well as a rather strong background staining in the von Willebrand factor IHC (Figure 3b). Cells lining the cavities and stromal cells were negative for the pan cytokeratin marker MNF116 (not shown).

The above described histologic and immunohistochemical features resemble previously described vascular anomalies in calves [7,8]. In spite of the often conflicting nomenclature terminology [6,7], a final diagnosis of multiple cutaneous, subcutaneous, and visceral vascular hamartomas compatible with a generalized form of BJA as described by Watson and Thompson [6] was made in this case.

### 3.3. Genetic Analysis

Assuming a spontaneous mutation as etiology for this most likely congenital condition, the sequencing of the whole genome of the affected calf was carried out. Filtering of the obtained variant catalogue for private variants exclusively present in the BJA-affected calf and absent in 496 available control genomes identified 31 private protein-changing variants, and subsequent visual inspection using IGV software confirmed 29 as real variants. Analyzing the occurrence of these variants in the global control cohort of 4110 genomes of a variety of breeds [28], six heterozygous protein-changing variants exclusively present in the genome of the affected calf were identified (Table S2). A total of 137 sequenced genomes from Simmental cattle were considered during variant filtering. Results of the functional impact prediction of these six heterozygous protein-changing variants are presented in Table 1.

Based on the function of the identified genes and in the predicted impact on the protein, the variants identified in *PREX1*, *UBE3B*, *ZSWIM6*, and *PCDHGA2* were considered the most likely candidate pathogenic variants for the observed phenotype. Unfortunately, no biological samples of the sire and dam were available to evaluate if one of these variants has occurred de novo in the calf.

No evidence for chromosomal abnormalities were detected by analyzing the obtained read depth or coverage along all chromosomes.

## 4. Discussion

We performed a comprehensive clinical, pathological, and genetic investigation in a Simmental calf displaying multifocal subcutaneous and visceral vascular hamartomas compatible with a generalized form of BJA.

We evaluated the hypothesis of a spontaneous mutation as the possible cause for this most likely congenital phenotype. Analysis of the genome sequence revealed six heterozygous protein-changing variants in the *PREX1*, *UBE3B*, *PCDHGA2, ZSWIM6*, *NR1H3*, and *C23H6orf132* genes that were exclusively present in the genome of the affected calf and absent in a global control cohort of more than 4500 cattle genomes of a variety of breeds. Therefore, we considered these apparently rare coding variants as possible candidates for the observed BJA phenotype. In the following, literature and in silico effect predictions are used to discuss a conceivable causal role. The missense variants identified in the *NR1H3* and *C23H6orf132* genes were not considered as putative causes for these vascular malformations because they were predicted to have a neutral or benign effect.

However, the missense variant found in *PREX1* was predicted to be deleterious by different tools such as PROVEAN and PredictSNP1 [34,36]. This gene belongs to the family of Rac guanine nucleotide exchange factors (Rac-GEF) and is activated by phosphatidylinositol 3,4,5-trisphosphate (PI (3,4,5) P3), which is generated by class I phosphoinositide 3-kinase (*PI3K*) and the β-gamma subunits of the heterotrimeric-G proteins (Gβγ) [38]. Furthermore, *PREX1* has an important role in the control of many fundamental cellular functions, including cell migration, actin cytoskeletal rearrangement, adhesion, and the production of reactive oxygen species (ROS) [39]. Additionally, the major effector of PREX1 protein activity is related to the induction of actin-mediated membrane ruffling and lamellipodia production at the leading edge of cell migration, and abnormally activated Rac is involved in the metastasis and invasion of tumor cells [40]. Evidence suggested that Rac and PREX1 protein are increased in cell proliferation and migration in several human cancers such as melanoma [41], breast cancer [42], prostate cancer [43], and oral squamous cell carcinoma [44]. However, no association between *PREX1* and the occurrence of vascular tumors has been reported to date.

The missense variant in *UBE3B* was predicted to be deleterious by PredictSNP1 [36]. An independent splice site variant in bovine *UBE3B* is associated with an autosomal-recessive inherited disorder called PIRM syndrome in Finnish Ayrshire cattle, which causes intellectual disability, retarded growth, and mortality (OMIA 001934-9913) [45], and which resembles the human autosomal-recessive Kaufman oculocerebrofacial syndrome (OMIM 244450) [46]. In addition, a recent study demonstrated that suppression of the E3 ubiquitin ligase UBE3B-mediated MYC ubiquitination and degradation caused by the integration of *TRIB3* with *MYC* is associated with high proliferation and self-renewal of lymphoma cells [47]. However, in the case presented in this study, the identified variant in *UBE3B* was heterozygous, and the calf did not show a phenotype compatible with these disorders.

Moreover, a disruptive in-frame deletion in *PCDHGA2* was predicted to have a deleterious impact in the protein using PROVEAN [34]. This gene presents a probability of loss-of-function intolerance score (pLI) of zero according to Genome Aggregation Database (gnomAD) [37]. Considering that transcripts with a pLI superior or equal to 0.9 are predicted to be loss-of-function (LoF) intolerant due to haploinsufficiency of the gene [48], *PCDHGA2* most likely does not belong to the group of LoF haploinsufficient genes. In addition, PCDHGA2 protein is a potential calcium-dependent cell-adhesion protein that might be involved in the establishment and maintenance of specific neuronal connections in the brain [49,50]. In humans, somatic mutations in *PCDHGA2* have been associated with cell and biological adhesion in aggressive papillary thyroid microcarcinomas [51]. However, and similarly to *PREX1*, no association between this gene and the occurrence of vascular tumors has been reported to date.

To date, no pathogenic variants of *ZSWIM6* have been reported in domestic animals. Interestingly, recent data obtained from human genome sequencing studies presented in the gnomAD [37] showed that the pLI for this gene was 1, meaning that *ZSWIM6* falls into the class of LoF haploinsufficient genes. The encoded protein, so-called Zinc finger SWIM domain-containing protein 6, is a protein of unknown function that is involved in the nervous system development and regulation [52]. Moreover, *ZSWIM6* enables the zinc ion binding [53] and is part of the Cul2-RING ubiquitin ligase complex [49]. In humans, pathogenic variants in the *ZSWIM6* gene (OMIM 615951) are associated to acromelic frontonasal dysostosis [54] and neurodevelopmental disorder with movement abnormalities, abnormal gait, and autistic features [52,55]. Additionally, the ZSWIM6 protein, according to the Biological General Repository for Interaction Datasets (BioGRID), is predicted to interact physically with ARIH1 [56], GLMN [57], HECW2 [58], HNRNPH1 [59] and HNRNPL [60]. Similarly to ZSWIM6, the GLMN is part of the Cul2-RING ubiquitin ligase complex [61] and known to be involved in numerous different processes, namely the normal development of the vasculature [62]. Particularly, autosomal dominant pathogenic variants in the *GLMN* gene (OMIM 138000) are associated with the development of glomuvenous malformations (GVMs) [63] and Blue rubber bleb nevus syndrome (BRBNS) (OMIM 112200) [64] in humans.

Based on these findings, we speculate whether the above-described vascular lesions in this calf could be due to an impaired interaction between ZSWIM6 and GLMN. However, it cannot be excluded that the phenotype displayed by this calf is due to one of the other remaining variants found exclusively in the sequenced case, or even to a combination of these. Either way, we hypothesize that one of these variants either occurred post-zygotically during the fetal development of the affected calf or represent a germline mutation that occurred in one of the parents. To prove that the private variants occurred was indeed de novo, genotyping of the sire and dam would be needed. Unfortunately, no samples from these animals were available at the time the genetic analysis was performed, therefore this hypothesis cannot be confirmed.

We hope that these findings may contribute to a better knowledge and characterization of BJA. However, it is highly unlikely that the candidate causal variants identified in the genome of the studied calf are responsible for other BJA cases since this condition encompasses several kinds of vascular anomalies, including both vascular malformations and tumors [6].

## 5. Conclusions

This report highlights the utility of WGS-based precision diagnostics for understanding the underlying genetics of rare disorders in animals with an available reference genome sequence and the value of surveillance for harmful genetic disorders in cattle breeding populations.

## Figures and Tables

**Table 1:** Pathogenicity prediction results for the six heterozygous protein-changing variants exclusively present in the genome of the BJA-affected calf and absent in global control cohort of more than 4500 genomes of a variety of breeds.

Gene	Effect	Protein-Changing	pLI ^1^	PROVEAN Score	PROVEAN Impact	MutPred2 Score	MutPred2 Impact	PredictSNP1 Score	PredictSNP1 Impact
*PREX1*	missense	p.Arg401Cys	1	−5.149	deleterious	0.387	neutral	0.719	deleterious
*UBE3B*	missense	p.Ala32Val	0	−1.946	neutral	0.583	neutral	0.510	deleterious
*PCDHGA2*	disruptive in-frame deletion	p.Lys141_Val142del	0	−11.366	deleterious	0.338	neutral	NA	NA
*ZSWIM6*	disruptive in-frame deletion	p.Ala146_Gly148del	1	1.280	neutral	0.366	neutral	NA	NA
*NR1H3*	missense	p.Thr46Met	0.9	−0.371	neutral	0.086	neutral	0.653	neutral
*C23H6orf132*	missense	p.Gly692Glu	NA	−0.907	neutral	0.035	neutral	0.826	neutral

^1^ probability of loss-of-function intolerance score (pLI) provided by the Genome Aggregation Database (gnomAD) [37]. NA, not available.

## Data Availability

The whole-genome data of the affected calf is freely available at the European Nucleotide Archive (ENA) under sample accession number SAMEA6528880.

## Supplementary Materials

The following are available online at https://www.mdpi.com/2076-2615/11/3/624/s1, Table S1: Project and Sample ID for public access of the whole genome sequenced genomes used in this study. EBI Accession numbers of all publicly available genome sequences. We compared the genotypes of the calf with 496 cattle genomes of various breeds that had been sequenced in the course of other ongoing studies and that were publicly available, Table S2: List of the remaining variants after the comparison to the global control cohort of 4110 genomes of other breeds (1000 Bull Genomes Project run 8; www.1000bullgenomes.com), revealing 6 protein-changing variants with a predicted moderate impact only present in the affected calf.

## References

[1] Alamo L., Beck-Popovic M., Gudinchet F., Meuli R. (2011). Congenital tumors: Imaging when life just begins. Insights Imaging.

[2] Wassef M., Blei F., Adams D., Alomari A., Baselga E. (2015). Vascular Anomalies Classi fi cation: Recommendations From the International Society for the Study of Vascular Anomalies. Pediatrics.

[3] ISSVA Classification of Vascular Anomalies. issva.org/classification.

[4] Mansfield S.A., Williams R.F., Iacobas I. (2020). Vascular tumors. Semin. Pediatr. Surg..

[5] Priestnall S.L., De Bellis F., Bond R., Alony-Gilboa Y., Summers B.A. (2010). Spontaneous regression of congenital cutaneous hemangiomas in a calf. Vet. Pathol..

[6] Watson T.D., Thompson H. (1990). Juvenile bovine angiomatosis: A syndrome of young cattle. Vet. Rec..

[7] Rösti L., Lauper J., Merhof K., Gorgas D., Ross S., Grest P., Welle M. (2013). Angeborene Gefässanomalien in der Maulhöhle bei zwei Kälbern. Schweiz Arch Tierheilkd.

[8] Sheahan B.J., Donnelly W. (1981). Vascular hamartoma in the gingiva of two neonatal calves. J. Am. Vet. Med. Assoc..

[9] Yeruham I., Abramovitch I., Perl S. (2004). Gingival vascular hamartoma in two calves. Aust. Vet. J..

[10] Stanton M., Meunier P., Smith D. (1984). Vascular hamartoma in the gingiva of two neonatal calves. J. Am. Vet. Med. Assoc..

[11] Wilson R.B. (1990). Gingival vascular hamartoma in three calves. J. Vet. Diagnostic Investig..

[12] Yeruham I., Perl S., Orgad U. (1999). Congenital skin neoplasia in cattle. Vet. Dermatol..

[13] Brisville A.C., Buczinski S., Chénier S., Francoz D. (2012). A cardiac vascular hamartoma in a calf: Ultrasonographic and pathologic images. J. Vet. Cardiol..

[14] Roth L., Bradley G.A. (1991). Pulmonary hamartoma in a calf. J. Comp. Pathol..

[15] Misdorp W. (2002). Tumours in calves: Comparative aspects. J. Comp. Pathol..

[16] Tontis A. (1994). Kongenitales kavernöses Hämangiom beim Brown-Swiss-Kalb, ein seletenes orales Blastom. Tierarztl. Prax..

[17] Baker J.C., Hultgren B.D., Larson V.L. (1982). Disseminated cavernous hemangioma in a calf. J. Am. Vet. Med. Assoc..

[18] Poulsen K., McSloy A., Perrier M., Prichard M., Steinberg H., Semrad S. (2008). Primary mandibular hemangiosarcoma in a bull. Can. Vet. J..

[19] Stock M.L., Smith B.I., Engiles J.B. (2011). Disseminated hemangiosarcoma in a cow. Can. Vet. J..

[20] Badylak S.F. (1983). Congenital Multifocal Hemangiosarcoma in a Stillborn Calf. Vet. Pathol..

[21] Hendrick M.J. (2016). Mesenchymal Tumors of the Skin and Soft Tissues. Tumors Domest. Anim..

[22] Cotchin E., Swarbrick O. (1963). Bovine cutaneous angiomatosis: A lesion resembling human pyogenic granuloma (granuloma telangiecticum). Vet. Rec..

[23] Lombard C., Levesque L. (1964). H’emangiomatose hyperplasique cutan’ee de vaches Normandes. Ann. Anat. Pathol. (Paris).

[24] Moulton J.E. (1978). Tumors in Domestic Animals.

[25] Waldvogel A., Hauser B. (1983). “Bovine kutane Angiomatose” in der Schweiz. Schweiz. Arch. Tierheilkd..

[26] Schilcher F., Gasteiner J., Palmetshofer G., Baumgartnerm W. (2000). Generalisierte juvenile Angiomatose bei einem Kalb. Wien. Tierarztl. Monatsschr..

[27] Rosen B.D., Bickhart D.M., Schnabel R.D., Koren S., Elsik C.G., Tseng E., Rowan T.N., Low W.Y., Zimin A., Couldrey C. (2020). De novo assembly of the cattle reference genome with single-molecule sequencing. Gigascience.

[28] Hayes B.J., Daetwyler H.D. (2019). 1000 Bull Genomes Project to Map Simple and Complex Genetic Traits in Cattle: Applications and Outcomes. Annu. Rev. Anim. Biosci..

[29] Chen S., Zhou Y., Chen Y., Gu J. (2018). Fastp: An ultra-fast all-in-one FASTQ preprocessor. Bioinformatics.

[30] Häfliger I.M., Wiedemar N., Švara T., Starič J., Cociancich V., Šest K., Gombač M., Paller T., Agerholm J.S., Drögemüller C. (2020). Identification of small and large genomic candidate variants in bovine pulmonary hypoplasia and anasarca syndrome. Anim. Genet..

[31] Robinson J.T., Thorvaldsdóttir H., Wenger A.M., Zehir A., Mesirov J.P. (2017). Variant review with the integrative genomics viewer. Cancer Res..

[32] Li H. (2011). A statistical framework for SNP calling, mutation discovery, association mapping and population genetical parameter estimation from sequencing data. Bioinformatics.

[33] R Core Team (2013). R: A Language and Environment for Statistical Computing.

[34] Choi Y., Chan A.P. (2015). PROVEAN web server: A tool to predict the functional effect of amino acid substitutions and indels. Bioinformatics.

[35] Pejaver V., Urresti J., Lugo-Martinez J., Pagel K., Lin G.N., Nam H.-J., Mort M., Cooper D., Sebat J., Iakoucheva L. (2017). MutPred2: Inferring the molecular and phenotypic impact of amino acid variants. Nat. Commun..

[36] Bendl J., Stourac J., Salanda O., Pavelka A., Wieben E.D., Zendulka J., Brezovsky J., Damborsky J. (2014). PredictSNP: Robust and Accurate Consensus Classifier for Prediction of Disease-Related Mutations. PLoS Comput. Biol..

[37] Karczewski K.J., Francioli L.C., Tiao G., Cummings B.B., Alföldi J., Wang Q., Collins R.L., Laricchia K.M., Ganna A., Birnbaum D.P. (2020). The mutational constraint spectrum quantified from variation in 141,456 humans. Nature.

[38] Welch H.C.E., Coadwell W.J., Ellson C.D., Ferguson G.J., Andrews S.R., Erdjument-Bromage H., Tempst P., Hawkins P.T., Stephens L.R. (2002). P-Rex1, a PtdIns(3,4,5)P3- and gβγ-regulated guanine-nucleotide exchange factor for Rac. Cell.

[39] Jaffe A.B., Hall A. (2005). Rho GTPases: Biochemistry and biology. Annu. Rev. Cell Dev. Biol..

[40] Vigil D., Cherfils J., Rossman K.L., Der C.J. (2010). Ras superfamily GEFs and GAPs: Validated and tractable targets for cancer therapy?. Nat. Rev. Cancer.

[41] Ryan M.B., Finn A.J., Pedone K.H., Thomas N.E., Der C.J., Cox A.D. (2016). ERK/MAPK signaling drives overexpression of the rac-GEF, PREX1, in BRAF-and NRAS-mutant melanoma. Mol. Cancer Res..

[42] Sosa M.S., Lopez-Haber C., Yang C., Wang H.B., Lemmon M.A., Busillo J.M., Luo J., Benovic J.L., Klein-Szanto A., Yagi H. (2010). Identification of the Rac-GEF P-Rex1 as an Essential Mediator of ErbB Signaling in Breast Cancer. Mol. Cell.

[43] Goel H.L., Pursell B., Shultz L.D., Greiner D.L., Brekken R.A., Vander Kooi C.W., Mercurio A.M. (2016). P-Rex1 Promotes Resistance to VEGF/VEGFR-Targeted Therapy in Prostate Cancer. Cell Rep..

[44] Wan S.C., Wu H., Li H., Deng W.W., Xiao Y., Wu C.C., Yang L.L., Zhang W.F., Sun Z.J. (2020). Overexpression of PREX1 in oral squamous cell carcinoma indicates poor prognosis. J. Mol. Histol..

[45] Venhoranta H., Pausch H., Flisikowski K., Wurmser C., Taponen J., Rautala H., Kind A., Schnieke A., Fries R., Lohi H. (2014). In frame exon skipping in UBE3B is associated with developmental disorders and increased mortality in cattle. BMC Genom..

[46] Flex E., Ciolfi A., Caputo V., Fodale V., Leoni C., Melis D., Bedeschi M.F., Mazzanti L., Pizzuti A., Tartaglia M. (2013). Loss of function of the E3 ubiquitin-protein ligase UBE3B causes kaufman oculocerebrofacial syndrome. J. Med. Genet..

[47] Li K., Wang F., Yang Z.N., Zhang T.T., Yuan Y.F., Zhao C.X., Yeerjiang Z., Cui B., Hua F., Lv X.X. (2020). TRIB3 promotes MYC-associated lymphoma development through suppression of UBE3B-mediated MYC degradation. Nat. Commun..

[48] Lek M., Karczewski K.J., Minikel E.V., Samocha K.E., Banks E., Fennell T., O’Donnell-Luria A.H., Ware J.S., Hill A.J., Cummings B.B. (2016). Analysis of protein-coding genetic variation in 60,706 humans. Nature.

[49] Gaudet P., Livstone M.S., Lewis S.E., Thomas P.D. (2011). Phylogenetic-based propagation of functional annotations within the Gene Ontology consortium. Brief. Bioinform..

[50] Uniprot UniProtKB - Q9Y5H1. https://www.uniprot.org/uniprot/Q9Y5H1.

[51] Song J., Wu S., Xia X., Wang Y., Fan Y., Yang Z. (2018). Cell adhesion-related gene somatic mutations are enriched in aggressive papillary thyroid microcarcinomas 06 Biological Sciences 0604 Genetics. J. Transl. Med..

[52] Tischfield D.J., Saraswat D.K., Furash A., Fowler S.C., Fuccillo M.V., Anderson S.A. (2017). Loss of the neurodevelopmental gene Zswim6 alters striatal morphology and motor regulation. Neurobiol. Dis..

[53] InterPro Zinc finger, SWIM-type (IPR007527). http://www.ebi.ac.uk/interpro/entry/InterPro/IPR007527/.

[54] Twigg S.R.F., Ousager L.B., Miller K.A., Zhou Y., Elalaoui S.C., Sefiani A., Bak G.S., Hove H., Hansen L.K., Fagerberg C.R. (2016). Acromelic frontonasal dysostosis and ZSWIM6 mutation: Phenotypic spectrum and mosaicism. Clin. Genet..

[55] Palmer E.E., Kumar R., Gordon C.T., Shaw M., Hubert L., Carroll R., Rio M., Murray L., Leffler M., Dudding-Byth T. (2017). A Recurrent De Novo Nonsense Variant in ZSWIM6 Results in Severe Intellectual Disability without Frontonasal or Limb Malformations. Am. J. Hum. Genet..

[56] Scott D.C., Rhee D.Y., Duda D.M., Kelsall I.R., Olszewski J.L., Paulo J.A., de Jong A., Ovaa H., Alpi A.F., Harper J.W. (2016). Two Distinct Types of E3 Ligases Work in Unison to Regulate Substrate Ubiquitylation. Cell.

[57] Huttlin E.L., Bruckner R.J., Paulo J.A., Cannon J.R., Ting L., Baltier K., Colby G., Gebreab F., Gygi M.P., Parzen H. (2017). Architecture of the human interactome defines protein communities and disease networks. Nature.

[58] Lu L., Hu S., Wei R., Qiu X., Lu K., Fu Y., Li H., Xing G., Li D., Peng R. (2013). The HECT type ubiquitin ligase NEDL2 is degraded by anaphase-promoting complex/cyclosome (APC/C)-Cdh1, and its tight regulation maintains the metaphase to anaphase transition. J. Biol. Chem..

[59] Uren P.J., Bahrami-Samani E., de Araujo P.R., Vogel C., Qiao M., Burns S.C., Smith A.D., Penalva L.O.F. (2016). High-throughput analyses of hnRNP H1 dissects its multi-functional aspect. RNA Biol..

[60] Fei T., Chen Y., Xiao T., Li W., Cato L., Zhang P., Cotter M.B., Bowden M., Lis R.T., Zhao S.G. (2017). Genome-wide CRISPR screen identifies HNRNPL as a prostate cancer dependency regulating RNA splicing. Proc. Natl. Acad. Sci. USA.

[61] Tron A.E., Arai T., Duda D.M., Kuwabara H., Olszewski J.L., Fujiwara Y., Bahamon B.N., Signoretti S., Schulman B.A., DeCaprio J.A. (2012). The Glomuvenous Malformation Protein Glomulin Binds Rbx1 and Regulates Cullin RING Ligase-Mediated Turnover of Fbw7. Mol. Cell.

[62] Brouillard P., Boon L.M., Mulliken J.B., Enjolras O., Ghassibé M., Warman M.L., Tan O.T., Olsen B.R., Vikkula M. (2002). Mutations in a novel factor, glomulin, are responsible for glomuvenous malformations (“glomangiomas”). Am. J. Hum. Genet..

[63] Irrthum A., Brouillard P., Enjolras O., Gibbs N.F., Eichenfield L.F., Olsen B.R., Mulliken J.B., Boon L.M., Vikkula M. (2001). Linkage disequilibrium narrows locus for venous malformation with glomus cells (VMGLOM) to a single 1.48 Mbp YAC. Eur. J. Hum. Genet..

[64] Yin J., Qin Z., Wu K., Zhu Y., Hu L., Kong X. (2019). Rare Germline GLMN Variants Identified from Blue Rubber Bleb Nevus Syndrome Might Impact mTOR Signaling. Comb. Chem. High Throughput Screen..

